# Serial RV wall stress measurements: association with right ventricular function in repaired Tetralogy of Fallot patients

**DOI:** 10.3389/fcvm.2023.1256792

**Published:** 2023-10-19

**Authors:** Savine C. S. Minderhoud, Alexander Hirsch, Francesca Marin, Isabella Kardys, José F. Rodríguez-Matas, Claudio Chiastra, Jolien W. Roos-Hesselink, Jolanda J. Wentzel, Willem A. Helbing, Ali C. Akyildiz

**Affiliations:** ^1^ Department of Cardiology, Erasmus MC, University Medical Center Rotterdam, Rotterdam, Netherlands; ^2^ Department of Radiology and Nuclear Medicine, Erasmus MC, University Medical Center Rotterdam, Rotterdam, Netherlands; ^3^ Laboratory of Biological Structure Mechanics (LaBS), Department of Chemistry, Materials and Chemical Engineering “Giulio Natta”, Politecnico di Milano, Milan, Italy; ^4^ PoliTo^BIO^Med Lab, Department of Mechanical and Aerospace Engineering, Politecnico di Torino, Turin, Italy; ^5^ Division of Paediatric Cardiology, Department of Pediatrics, Erasmus MC, University Medical Center Rotterdam, Rotterdam, Netherlands; ^6^ Division of Pediatric Cardiology, Department of Pediatrics, Radboud University Medical Center, Nijmegen, Netherlands; ^7^ Department of Biomechanical Engineering, Delft University of Technology, Delft, Netherlands

**Keywords:** Tetralogy of Fallot, right ventricle, cardiovascular magnetic resonance, computational modelling, wall stress

## Abstract

**Background:**

Optimal timing of pulmonary valve replacement (PVR) in Tetralogy of Fallot (TOF) patients remains challenging. Ventricular wall stress is considered to be an early marker of right ventricular (RV) dysfunction.

**Objectives:**

To investigate the association of RV wall stresses and their change over time with functional parameters in TOF patients.

**Methods:**

Ten TOF patients after surgical repair with moderate/severe pulmonary regurgitation were included. At two timepoints (median follow-up time 7.2 years), patient-specific computational biventricular models for wall stress assessment were created using CMR short-axis cine images and echocardiography-based RV pressures. RV ejection fraction (RVEF), NT-proBNP and cardiopulmonary exercise tests were used as outcome measures reflecting RV function. Associations between regional RV diastolic wall stress and RV function were investigated using linear mixed models.

**Results:**

Increased wall stress correlated with lower RV mass (*r*_rm _= −0.70, *p* = 0.017) and lower RV mass-to-volume (*r*_rm _= −0.80, *p* = 0.003) using repeated measures. Wall stress decreased significantly over time, especially in patients with a stable RVEF (*p* < 0.001). Higher wall stress was independently associated with lower RVEF, adjusted for left ventricular ejection fraction, RV end-diastolic volume and time since initial surgery (decrease of 1.27% RVEF per kPa increase in wall stress, *p* = 0.029) using repeated measurements. No association was found between wall stress, NT-proBNP, and exercise capacity.

**Conclusions:**

Using a computational method to calculate wall stress locally in geometrically complex ventricles, we demonstrated that lower wall stress might be important to maintain ventricular function. RV wall stress assessment can be used in serial follow-up, and is potentially an early marker of impending RV dysfunction.

## Introduction

Tetralogy of Fallot (TOF) is the most common cyanotic congenital heart defect. Long-term survival of 72% at 40 years after surgery has been reported (1). Heart failure and sudden cardiac death are the main causes of late mortality in these patients (1). Right ventricular (RV) function is an important parameter in this context as it is an independent predictor of mortality, sustained ventricular tachycardia and heart failure (2).

Various factors contribute to RV dysfunction in TOF patients, including longstanding pulmonary regurgitation (PR) and/or stenosis (3). With the initial repair, pulmonary stenosis is relieved. However, many patients are left with PR. The resulting chronic volume overload leads to progressive RV dilatation and dysfunction (4). Pulmonary valve replacement (PVR) is often needed and may improve patient's condition. PVR should be timed before irreversible RV dysfunction occurs, but should also be timed late enough to limit the number of re-interventions (3). Current indicators for PVR timing may lead to suboptimal post-operative results. Better markers to predict impending RV functional decline are needed (5). Previously, end-diastolic mechanical stress in the RV wall was suggested as a predictor for post-operative RV ejection fraction (EF) response (6), indicating that this mechanical marker might be an early indicator of RV dysfunction.

RV wall stress in chronic PR, changes in wall stress over time and how this relates to clinical end points is currently unknown. Accordingly, this study aims to investigate the change in RV wall stress over time in TOF patients with moderate or severe PR, and to assess the association of wall stress with functional parameters.

## Material and methods

### Study population

Repaired TOF patients with the following criteria were enrolled: (1) ≥moderate PR, (2) two sequential cardiovascular magnetic resonance (CMR) examinations with at least a time gap of four years in between and (3) echocardiography within one year from each CMR examination. Patients were selected from previous CMR studies (7–9). Patients with a RV outflow tract intervention between the CMRs were excluded. Patients underwent a comprehensive clinical assessment, NT-proBNP measurements, cardiopulmonary exercise test, CMR examination and echocardiography, as previously reported (7). Percentage of predicted work load and percent predicted peak rate of oxygen consumption (VO_2_) were variables used from the results of cardiopulmonary exercise tests. Local institutional ethics committee approved the study, and all patients provided written informed consent.

### CMR image acquisition and analysis

CMR images were acquired with clinical 1.5 T MRI scanners (GE Healthcare, Milwaukee, Wisconsin, USA). Included patients had undergone a CMR examination with at least a stack short-axis balanced steady-state free precession (bSSFP) cine images and phase contrast (PC) imaging of the pulmonary artery. Using a retrospective ECG-gated bSSFP sequence during mild end-expiratory breath-hold, ten to fourteen contiguous short-axis images were acquired covering the left ventricle (LV) and RV from basis to apex. Typical scan parameters were field of view(FOV) 30–38 cm, phase FOV 75%–100%, slice thickness of 6–8 mm with an interslice gap of 2–4 mm, matrix size 192 × 160 and 24 reconstructed phases per cardiac cycle (7). Breath-hold, retrospective ECG-gated through-plane PC imaging was planned perpendicular to the main pulmonary artery approximately 1 cm distal of the valve and before the pulmonary artery bifurcation. VENC was set at 180 cm/s and increased if necessary (7). Directly after the CMR examination, the exact same PC sequence parameters were used to scan a static gel phantom for phase off set error correction.

In Qmass (Medis, Leiden, The Netherlands), LV and RV endo- and epicardial contours were drawn on short axis CMR images during end-systole and end-diastole by one observer (SCSM) and were reviewed by a second observer (AH). Based on these contours, LV and RV end-diastolic volumes, end-systolic volumes and EF were calculated. RV mass was determined in end-systole {[epicardial volume–endocardial volume (in ml)] × 1.05 g/ml}. Volumes and mass were corrected for body surface area. With QFlow (Medis, Leiden, The Netherlands), PR fraction and regurgitation volume were quantified using PC imaging. Flow measurements were obtained after background subtraction with a static gel phantom.

### Echocardiography

RV diastolic pressure was derived based on the inferior vena cava diameter (≤20 mm or >20 mm) and percentage of collapse (≤50% or >50) on echocardiography (10). The maximum peak gradient across the pulmonary valve was determined using Doppler measurements and calculated using the modified Bernoulli equation [ΔP = 4 × (V_max_)^2^].

### Wall stress analysis

**Figure 1:**
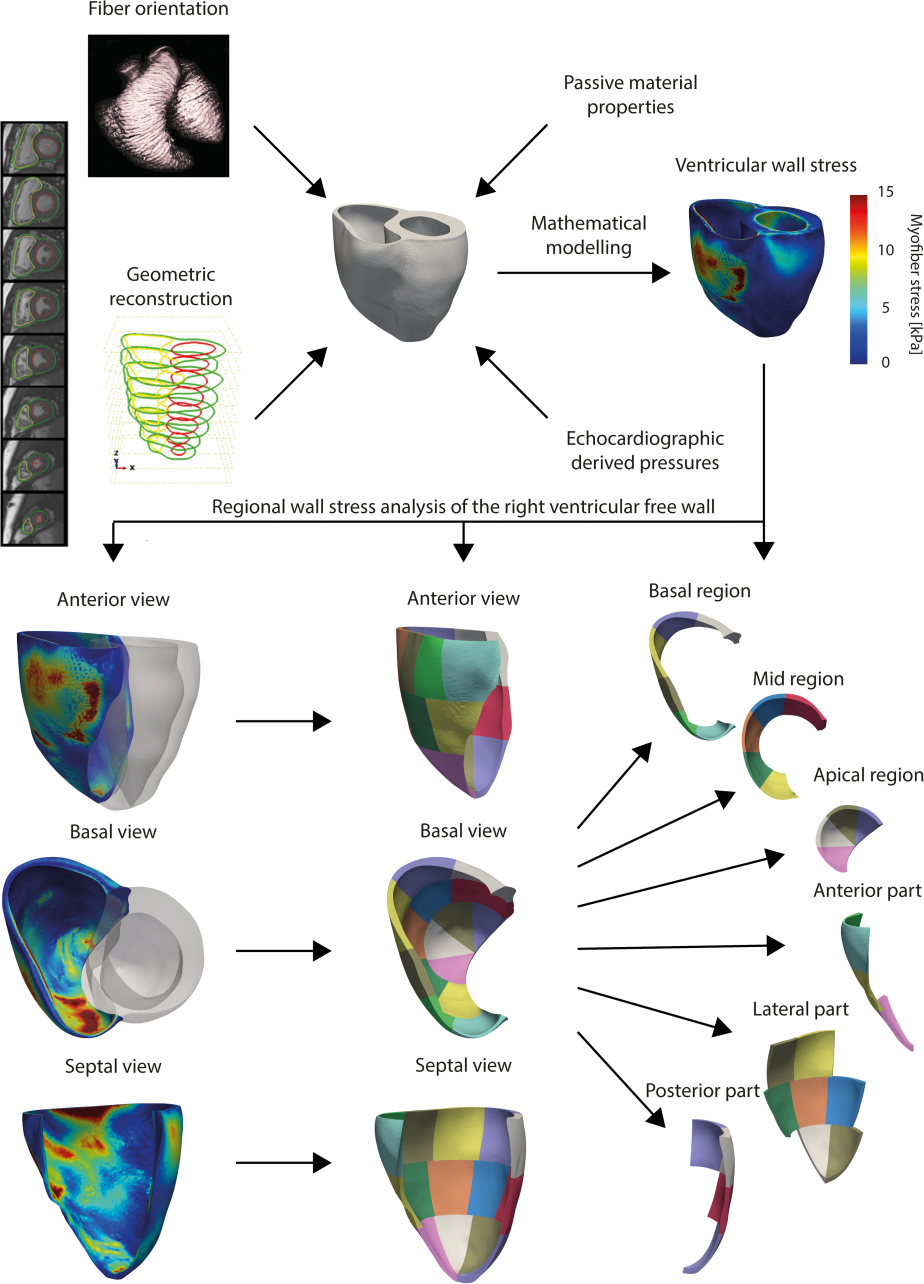
CMR-based finite element cardiac modelling workflow. Three-dimensional geometric reconstruction was obtained through volumetric interpolation of stacked short axis contours. Fibre orientation, passive material properties and right ventricular diastolic pressure were implemented. With finite element analysis, ventricular wall stresses were calculated. Right ventricular free wall was divided in fifteen segments.

Diastolic wall stress and strain were calculated using finite element (FE) analysis performed with Abaqus/Standard (Dassault Systèmes Simulia Corp., Providence, RI, USA). The stack of short axis CMR contours was interpolated to create three-dimensional biventricular models (Figure 1). The LV and RV walls were assumed to be hyperelastic, incompressible and anisotropic (11) with material parameters taken from Wang et al. (12). A smooth transmural variation of the fibre orientation from −70° at the epicardium to +80° at the endocardium was assumed as described by Wong et al. and implemented in the computational models (13).

Boundary and loading conditions were prescribed to the FE models as follows. Biventricular computational models were restrained from movement in the basal plane, simulating a stiff valvular plane. No constrains were applied to the apex that could move freely. Diastolic filling of the RV and LV was simulated by prescribing pressures on the ventricular endocardial surfaces. RV diastolic pressure estimate was obtained from echocardiography and assigned to the RV, while a physiological pressure of 9 mmHg was assigned to the LV.

These three-dimensional, mechanical FE models were used to simulate the passive filling during diastole. In total, twenty FE models were generated for ten patients at two timepoints. The end-diastolic wall stress (maximum principal wall stress) and distributions of stress in the RV free wall were computed. Maximum principal stress is the maximum values of wall stress in any direction. Mesh independency of the FE results was confirmed by mesh convergence analysis.

For the analysis, the septal part of the RV wall was excluded, as the ventricular septum is considered to belong morphologically to the LV (Figure 1). Because of regional variation of the magnitude of wall stress, a regional analysis approach was chosen. A standardized segmentation method for analysis of the LV was adapted and applied to the free wall of RV (Figure 1) (14), using a previously proposed method to create fifteen segments of approximately equal size. First, the RV free wall was divided into apical, mid and basal regions, which were further subdivided into four, five and six segments respectively (15) (Figure 1). These fifteen segments were also used to make a second division of the RV free wall into an anterior, lateral and posterior part (Figure 1). The mean and 95th-percentile peak wall stresses were calculated per region. The weighted mean, defined as the mean of the fifteen regional values, and peak RV wall stresses per biventricular model were also calculated.

### Statistical analysis

Continuous values were expressed as mean with standard deviation (SD) or, in case of skewed distribution, as median with a range or interquartile (IQR) range. Categorical data were summarized with frequencies and percentages. Regional wall stresses were log transformed because of a skewed distribution to calculate the geometric mean and geometric SD factor and are presented on the linear scale as geometric mean with range (geometric mean/geometric SD factor-geometric mean × geometric SD factor). Patients were divided in a group with a stable RVEF (RVEF follow-up minus RVEF baseline ≥0%) and a group with a decreasing RVEF (delta RVEF <0%) over time. RVEF was chosen because of its strong association with adverse clinical outcomes and exercise capacity in patients with repaired TOF (2, 16, 17). Continuous standard imaging parameters were compared with a Mann–Whitney test between groups and with a Wilcoxon one-sample test between baseline and follow-up.

Linear mixed models with a random intercept for each patient were used to investigate (1) local differences in wall stress (average and 95th percentile wall stress) between RV free wall regions (basal vs. mid vs. apical) and parts (anterior vs. lateral vs. posterior) (2) local change in wall stress between baseline and follow-up (3) local wall stress differences between patients with a stable RVEF and with a decreasing RVEF (4) associations of wall stress with clinical end-points [RVEF, NT-proBNP, exercise testing (peak VO_2_ and percentage of predicted work)] and (5) associations of baseline PR and PR volume with change of wall stress over time. To account for spatial autocorrelation between the wall stress regions, a spatial Gaussian correlation structure was used in the models 1–3 and 5, based on the x-, y- and z-coordinates of regions. Standardized x-, y- and z-coordinates of regions were used for comparison between baseline and follow-up (model 2) and between patients (stable vs. decreasing RVEF, model 3) and. All models, except model 1, were corrected for time since initial surgery (follow-up duration), by adding this variable as a fixed effect.

All dependent parameters were transformed using log2 when the residuals plots showed deviation from normality. For visualization purposes, correlation plots were created showing the weighted mean wall stress values. For correlations between variables that include both baseline and follow-up measurements (repeated measurements), we controlled for within-individual association using a repeated measurement correlation (rmcorr package in R) (18). All analyses have been performed in R Statistical Software version 4.1.0 (R Foundation for Statistical Computing, Vienna, Austria). Two-tailed *p*-values below 0.05 were considered statistically significant.

## Results

### Study population and clinical parameters

Ten patients were included in the study. Median age was 24 years (IQR 17–28) at baseline and five (50%) were males (Table 1). Median follow-up period between the baseline and follow-up CMR study was 7.2 years (IQR 6.9–7.5). At baseline, median RV end-diastolic volume was 140 ml/m^2^ (IQR 129–144), RVEF 46% (IQR 42–49), RV mass 15 g/m^2^ (IQR 12–18), RV mass-to-volume ratio 0.11 g/ml (IQR 0.09–0.13) and PR 41% (IQR 36–45). All patients had ≤mild tricuspid regurgitation during the both visits. Diastolic echocardiography-based RV pressures were estimated at 3 mmHg (IQR 3–3) in all patients at both timepoints. At follow-up, RV end-diastolic volume was 150 ml/m^2^ (IQR 144–151) and RVEF was 46% (IQR 44–48) (Table 2). Overall, none of the parameters changed significantly between baseline and follow-up (Table 2). In four patients, RVEF decreased between baseline and follow-up. In these patients, RV mass decreased more and a larger PR volume per beat was present at baseline (Table 2).

**Table 1:** Baseline characteristics (*n* = 10).

Male	5 (50%)
Age (years)	24 (17–28)
Height (cm)	172 (169–175)
Weight (kg)	67 (61–75)
BSA (m^2^)	1.78 (1.72–1.87)
Systolic blood pressure (mmHg)	119 (102–134)
Diastolic blood pressure (mmHg)	71 (63–81)
NYHA class I	10 (100%)
QRS duration (ms)	97 (112–143)
NT-proBNP (pmol/L)^a^	28 (12–39)
Age of initial repair (years)	0.5 (0–2.5)
Transannular patch, *n* (%)	9 (90%)
Follow-up period (years)^b^	7.2 (6.9–7.5)
Cardiovascular magnetic resonance	
LVEF (%)	54 (50–57)
RVESV indexed (ml/m^2^)	72 (75–79)
RVEDV indexed (ml/m^2^)	140 (129–144)
RVEF (%)	46 (42–49)
RV mass indexed (g/m^2^)	15 (12–18)
RV mass-to-volume ratio (g/ml)	0.11 (0.09–0.13)
PR fraction (%)	41 (36–45)
Pulmonary regurgitant volume (ml/beat)	48 (41–60)
Echocardiography	
Estimated RV diastolic pressure (mmHg)	3 (3–3)
V_max_ pulmonary valve (m/s)	2.0 (1.9–2.1)

Values are presented as median (IQR), number (percentage).

IQR, interquartile range; NYHA, New York heart association; NT-proBNP, N-terminal-pro brain B-type natriuretic peptide; LVEF, left ventricular ejection fraction; RVESV indexed, right ventricular end-systolic volume indexed to body surface area; RVEDV indexed, right ventricular end-diastolic volume indexed to body surface area; RV mass indexed, right ventricular mass indexed to body surface area; RVEF, right ventricular ejection fraction; RV, right ventricular; PR fraction, pulmonary regurgitation fraction.

^a^ Data of 8 patients.

^b^ Follow-up period between baseline and follow-up cardiovascular magnetic resonance.

**Table 2:** Comparison of imaging parameters between patients with a stable and decreasing right ventricular ejection fraction.

	Total (*n* = 10)	Patients with a stable RVEF (*n* = 6)	Patients with a decreasing RVEF (*n* = 4)	*p*-value*
Baseline				
RVEDV indexed (ml/m^2^)	140 (129–144)	132 (120–144)	147 (141–165)	0.171
RV mass indexed (g/m^2^)	15 (12–18)	13 (12–16)	18 (16–22)	0.067
RV mass-to-volume ratio (g/ml)	0.11 (0.09–0.13)	0.10 (0.09–0.11)	0.12 (0.10–0.15)	0.610
PR fraction (%)	41 (36–45)	38 (27–46)	41 (40–45)	0.519
PR volume (ml/beat)^a^	48 (41–60)	42 (32–47)	60 (51–76)	0.038
V_max_ pulmonary valve (m/s)	2.0 (1.9–2.1)	2.0 (1.7–2.2)	2.0 (1.9–2.0)	0.762
Follow-up				
RVEDV indexed (ml/m^2^)	150 (144–151)	144 (134–151)	152 (150–164)	0.114
RV mass indexed (g/m^2^)	17 (14–18)	16 (14–18)	16 (14–18)	0.476
RV mass-to-volume ratio (g/ml)	0.12 (0.10–0.13)	0.12 (0.12–0.13)	0.12 (0.09–0.13)	0.476
PR fraction (%)	47 (34–47)	43 (33–47)	48 (47–50)	0.067
PR volume (ml/beat)^a^	57 (36–83)	50 (39–71)	84 (83–84)	0.010
V_max_ pulmonary valve (m/s)	2.0 (1.7–2.1)	2.0 (1.7–2.1)	1.8 (1.7–2.0)	0.668
Changes over time				
Δ RVEDV indexed (ml/m^2^)	6 (4–8)	6 (6–8)	4 (−1–8)	0.610
Δ RV mass indexed (g/m^2^)	2 (0–2)	2 (2–5)	−1 (−2–0)	0.010
Δ RV mass-to-volume ratio (g/ml)	0.01 (−0.01–0.01)	0.01 (0.01–0.02)	−0.02 (−0.02–−0.02)	0.067
Δ PR fraction (%)	3 (−1–5)	−1 (−4–3)	6 (4–7)	0.054
Δ PR volume (ml/beat)^a^	1 (−4–13)	0 (−4–1)	23 (7–32)	0.257
Δ V_max_ pulmonary valve (m/s)	0.1 (−0.1–0.3)	0.1 (0.1–0.3)	−0.2 (−0.3–0.1)	0.197

Median (IQR).

EDV, end-diastolic volume; IQR, interquartile range; RV, right ventricular; PR fraction, pulmonary regurgitation fraction; PR volume, pulmonary regurgitant volume; V_max_, peak velocity.

^a^ PR volume is the backward regurgitant volume per beat.

* Mann–Whitney test.

### Mechanical wall stress, ventricular volumes and mass

**Figure 2:**
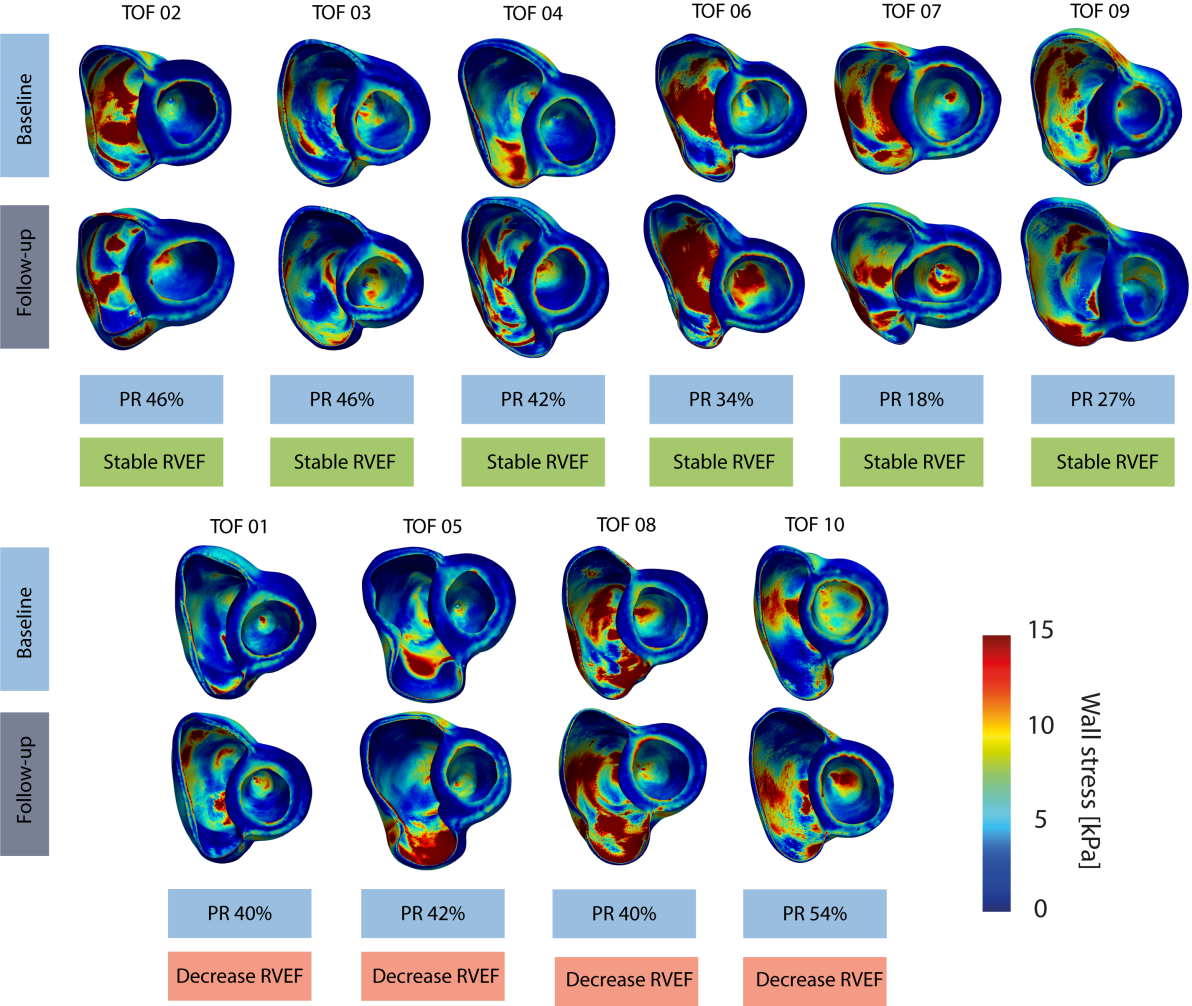
Overview of wall stress maps per patient. Top computational models refer to the baseline and bottom models refer to follow-up timepoint, stable RVEF indicates a stable RVEF over time and decrease RVEF indicates a decreasing RVEF over time. TOF, Tetralogy of Fallot.

**Figure 3:**
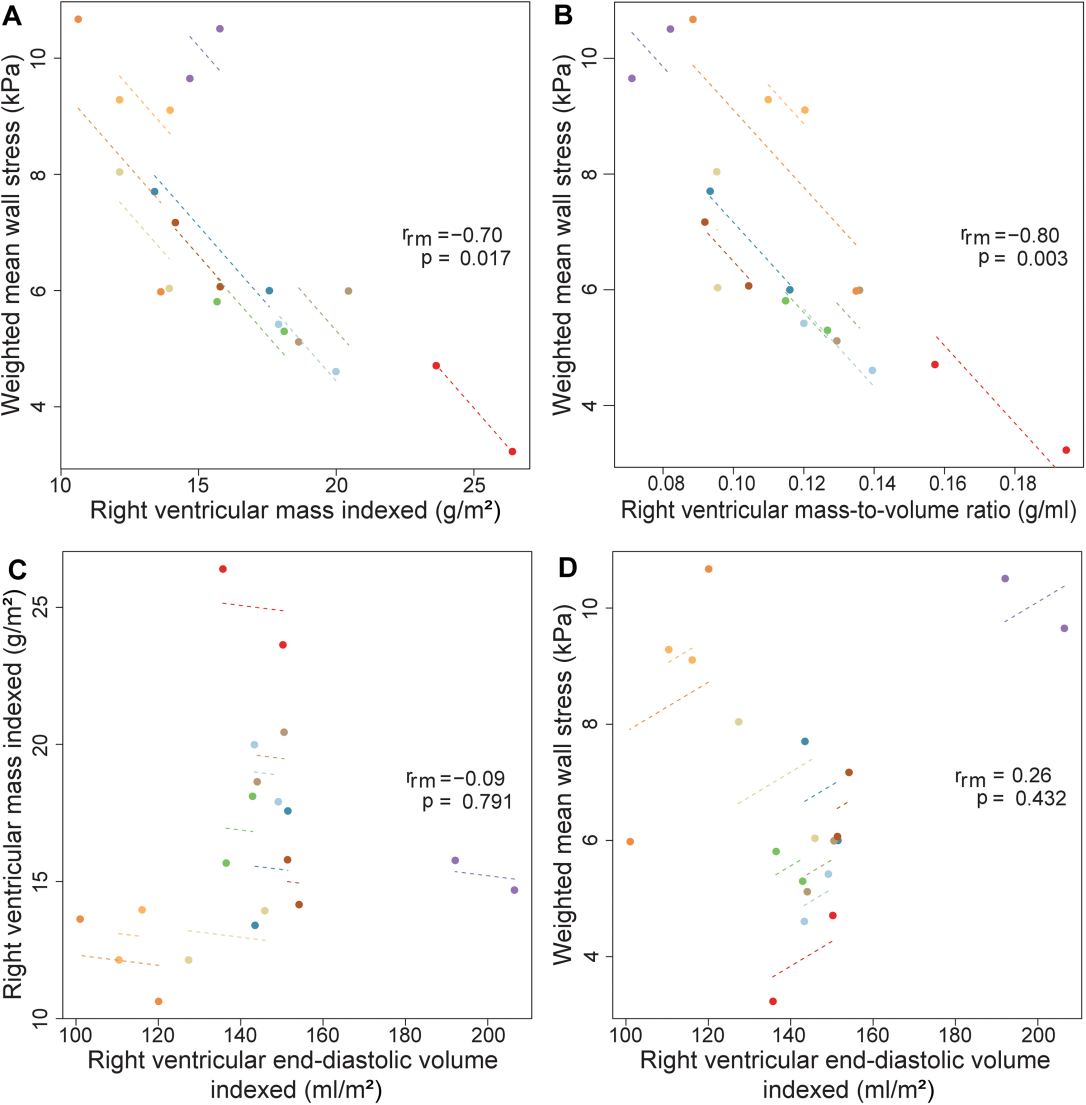
Correlations between wall stress, RV mass, volume and mass-to-volume ratio using repeated measurements. Repeated measures correlations for the relationship between (**A**) right ventricular mass indexed and weighted mean wall stress (**B**) right ventricular mass-to-volume ratio and weighted mean wall stress (**C**) right ventricular volume indexed and mass indexed (**D**) right ventricular end-diastolic volume indexed and average wall stress, measured two times in ten Tetralogy of Fallot patients. Longitudinal measurements and the correlation trend line are coloured per individual patient. *r*_rm_ is the correlation coefficient of within-individual association in repeated measures correlation, and the associated *p*-value is shown.

Figure 2 provides an overview of the regional wall stress distributions at baseline and follow-up per patient. Clear regional differences in wall stress patterns are observed within the RV, but also between baseline and follow-up and between patients. Weighted mean wall stress was 6.89 kPa (IQR 5.29–8.97) at baseline and 6.00 kPa (IQR 5.56–6.89) at follow-up and did not change significantly between baseline and follow-up (*p* = 0.460). Correlations between average wall stress, RV volumes and mass measured both at baseline and at follow-up are presented in Figure 3. Higher average wall stress was associated with lower RV mass (*r*_rm _= −0.70, *p* = 0.017, Figure 3A) and lower mass-to-volume ratio (*r*_rm _= −0.80, *p* = 0.003, Figure 3B). No associations were found between RV end-diastolic volume and mass and between RV end-diastolic volume and average wall stress (Figures 3C,D).

Overall, at baseline, regional analysis showed that wall stress in the mid region (7.24 ± 1.64 kPa, mean, SD) was higher compared to wall stress in the basal region {basal 5.40 ± 1.70 kPa, 0.37 difference [95% confidence interval (CI) 0.11, 0.63], *p* = 0.006} in log2 wall stress between mid and basal region), but not different from wall stress in the apical region [5.99 ± 1.70 kPa, 0.20 difference (95% CI −0.14, 0.53), *p* = 0.248] in log2 wall stress between mid and apical region) (Table 3). Furthermore, wall stress in the lateral part was significantly higher than wall stress in the posterior part [0.42 difference (95% CI 0.26, 0.58), *p* < 0.001] in log2 wall stress between lateral and posterior part), which was in turn significantly higher than wall stress in the anterior part [0.96 difference (95% CI 0.80, 1.12), *p* < 0.001] in log2 wall stress between lateral and posterior part) (Supplementary Table S1). The peak wall stress was also the highest in the mid region and lateral part (data not shown). Both at baseline and follow-up, no differences were observed in regional wall stress, RV end-diastolic volume, RV mass and RV mass-to-volume ratio between patients with a stable and decreasing RVEF (Tables 2, 3).

**Table 3:** Regional wall stress changes over time stratified according to right ventricular function.

	Total (*n* = 10)	Patients with a stable RVEF (*n* = 6)	Patients with a decreasing RVEF (*n* = 4)	β (95% CI)**	*P*-value**
Entire right ventricular free wall					
Number of regions	150	90	60		
Wall stress baseline (kPa)	6.12 (3.61–10.40)	6.91 (4.26–11.22)	5.11 (2.96–8.81)	0.43 (−0.36, 1.22)^a^	0.246
Wall stress follow-up (kPa)	5.84 (3.54–9.62)	5.58 (3.31–9.40)	5.98 (3.57–10.00)	0.04 (−0.66, 0.75)^a^	0.889
Change over time (kPa)	−0.41 ± 3.42	−1.39 ± 3.94	1.06 ± 1.55	−2.79 (−5.19, −0.38)	0.029
β (95% CI)^a,*^	−0.54 (−0.76, −0.31)	−0.53 (−0.68, −0.39)	−0.20 (−0.81, 0.42)	–	–
*P*-value baseline vs. FU*	<0.001	<0.001	0.530	–	–
Basal right ventricular free wall					
Number of regions	60	36	24		
Wall stress baseline (kPa)	5.40 (3.18–9.17)	5.87 (3.63–9.52)	4.77 (2.67–8.52)	0.16 (−0.64, 0.96)^a^	0.651
Wall stress follow-up (kPa)	5.34 (3.34–8.56)	5.12 (3.13–8.38)	5.70 (3.67–8.83)	−0.07 (−0.76, 0.62)^a^	0.823
Change over time (kPa)	−0.27 ± 2.88	−0.90 ± 3.43	0.67 ± 1.36	−1.22 (−3.33, 0.89)	0.294
β (95% CI)^a,*^	−0.20 (−0.48, 0.08)	−0.27 (−0.59, 0.06)	−0.12 (−0.77, 0.53)	–	–
*P*-value baseline vs. FU*	0.167	0.111	0.718	–	–
Mid right ventricular free wall					
Number of regions	50	30	20		
Wall stress baseline (kPa)	7.24 (4.43–11.85)	8.38 (5.45–12.88)	5.82 (3.50–9.68)	0.55 (−0.31, 1.42)^a^	0.174
Wall stress follow-up (kPa)	6.77 (4.35–10.53)	6.16 (4.12–9.21)	7.79 (4.85–12.49)	−0.24 (−1.13, 0.65)^a^	0.546
Change over time (kPa)	−0.65 ± 3.22	−2.49 ± 2.71	2.11 ± 1.46	−4.87 (−7.65, −2.08)	0.004
β (95% CI)^a,*^	−0.40 (−0.68, −0.13)	−0.66 (−0.93, −0.39)	0.06 (−0.55, 0.67)	–	–
*P*-value baseline vs. FU*	0.004	<0.001	0.848	–	–
Apical right ventricular free wall					
Number of regions	40	24	16		
Wall stress baseline (kPa)	5.99 (3.52–10.18)	6.92 (4.27–11.21)	4.82 (2.81–8.27)	0.56 (−0.51–1.64)^a^	0.257
Wall stress follow-up (kPa)	5.53 (3.11–9.85)	5.60 (2.86–11.01)	5.42 (3.61–8.16)	−0.11 (−0.97, 0.74)^a^	0.801
Change over time (kPa)	−0.33 ± 4.36	−0.77 ± 5.53	0.33 ± 1.30	−2.55 (−7.72, 2.61)	0.281
β (95% CI)^a,*^	−0.37 (−0.67, −0.07)	−0.38 (−0.68, −0.08)	−0.01 (−0.86, 0.83)	–	–
*P*-value baseline vs. FU*	0.016	0.013	0.974	–	–

Values are presented as numbers, mean and standard deviation (SD) or in case of a skewed distribution as geometric mean and geometric SD factor presented on the linear scale as geometric mean with range (geometric mean/geometric SD factor—geometric mean × geometric SD factor). Linear mixed effect models are created with wall stress as dependent variable and timepoint (baseline or follow-up) as independent variable adjusted for time since initial surgery. Models have a random intercept per patient and a spatial Gaussian correlation structure.

RVEF, right ventricular ejection fraction; FU, follow-up.

^a^ Results are presented as the mean difference with 95% confidence interval (CI) of the wall stress expressed as 2log kPa.

* Comparing baseline with follow-up.

** Comparing patients with a stable and a declining RVEF.

### Changes over time

Regional free wall stress of the RV decreased between baseline and follow-up [−0.54 change (95% CI −0.76, −0.31), *p* < 0.001, in log2 wall stress between baseline and follow-up]. While in patients with a decreasing RVEF, wall stress did not change significantly over time [β −0.20 (95% CI −0.81, 0.42), *p* = 0.530], in patients with a stable RVEF the wall stress decreased significantly [β −0.53 (95% CI −0.68, −0.39), *p* < 0.001]. The regional free wall stress decrease over time in patients with a stable RVEF was larger compared to that of patients with a decreasing RVEF [β −2.79 (95% CI −5.19, −0.38), *p* = 0.029] when corrected for follow-up period. The difference of the change of wall stress between the two groups was most prominent in the mid region [β −4.87 (95% CI −7.65, −2.08), *p* = 0.004] and anterior part [β −2.04 (95% CI −3.50, −0.58), *p* = 0.029]. Peak wall stress did not change over time, both in patients with a stable and with a decreasing RVEF.

Patients with a stable RVEF demonstrated a significant increase of RV mass and RV mass-to-volume ratio (both *p* = 0.031) between baseline and follow-up. These patients showed a larger RV mass increase during the follow-up period compared to patients with a decreasing RVEF (*p* = 0.010). Addionally, there was a tendency towards a larger RV mass-to-volume ratio decrease in patients with a decreasing RVEF compared to patients with a stable RVEF (*p* = 0.067) (Table 2). There were no significantly different changes in RV end-diastolic volume over time between patients with a decreasing RVEF and those with a stable RVEF.

### Functional outcomes and wall stress

**Figure 4:**
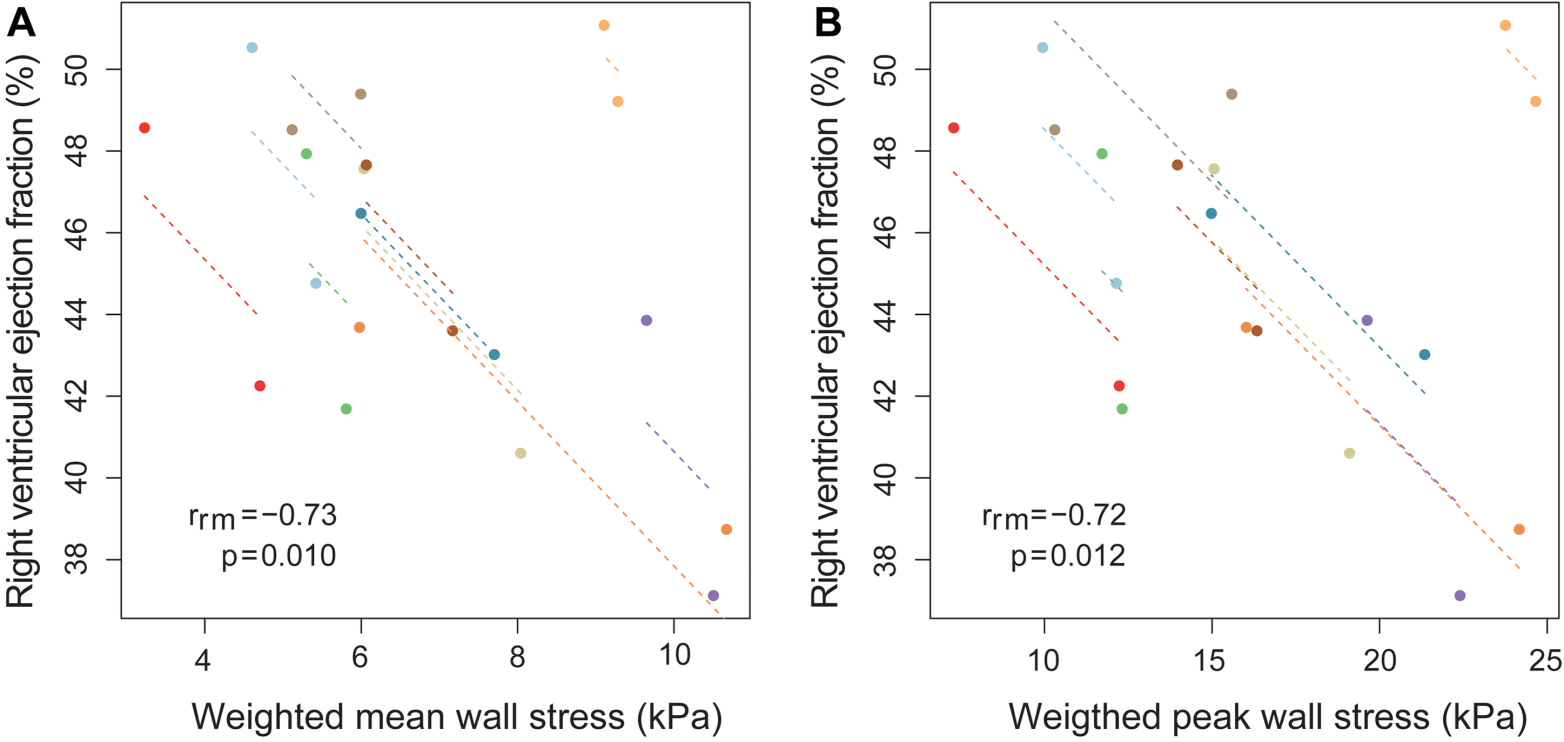
Association between RV ejection fraction and weighted mean wall stress using repeated measurements. Association between (**A**) weighted mean wall stress of the right ventricular (RV) free wall and RV ejection fraction (**B**) weighted peak wall stress of the RV free wall and RV ejection fraction, using repeated measurements at two timepoint in ten Tetralogy of Fallot patients. Longitudinal measurements and the correlation trend line are coloured per individual patient. *r*_rm_ is the correlation coefficient of within-individual association in repeated measures correlation, and the associated *p*-value is shown.

In Figure 4A, the relationship between weighted mean wall stress of the free wall and RVEF is depicted. Higher wall stress was associated with a lower RVEF [β −1.16% RVEF per kPa increase in wall stress (95% CI −2.12, −0.20), *p* = 0.022]. When also corrected for LVEF, RV end-diastolic volume and time since surgery, average wall stress remained independently associated with RVEF [β −1.27% RVEF per kPa increase in wall stress (95% CI −2.36, −0.18), *p* = 0.029] (Supplementary Table S2). Mass-to-volume ratio was not associated with RVEF (Supplementary Table S2). There was also a tendency towards a negative association between weighted peak wall stresses and RVEF [β −0.41% RVEF per kPa increase in wall stress (95% CI −0.83, 0.01), *p* = 0.055] (Figure 4B).

Wall stress and peak wall stress at baseline were both not associated with the RVEF at follow-up (wall stress β −0.15 (95% CI −1.49, 1.20), *p* = 0.810, peak wall stress β 0.06 (95% CI −0.46, 0.58), *p* = 0.805), corrected for follow-up duration. This was also the case when corrected for follow-up duration and RVEF at baseline.

At follow-up, the median NT-proBNP level was 22 pmol/L (IQR 16–43) and the median percentage of predicted work load was 92% (IQR 88–110). Wall stress at baseline was not associated with NT-proBNP and percentage of predicted work load at follow-up when corrected for follow-up period. Peak VO_2_ was available at follow-up for only three patients. Therefore, analyses of this parameter as end-point were not performed.

### Pulmonary regurgitation and wall stress

**Figure 5:**
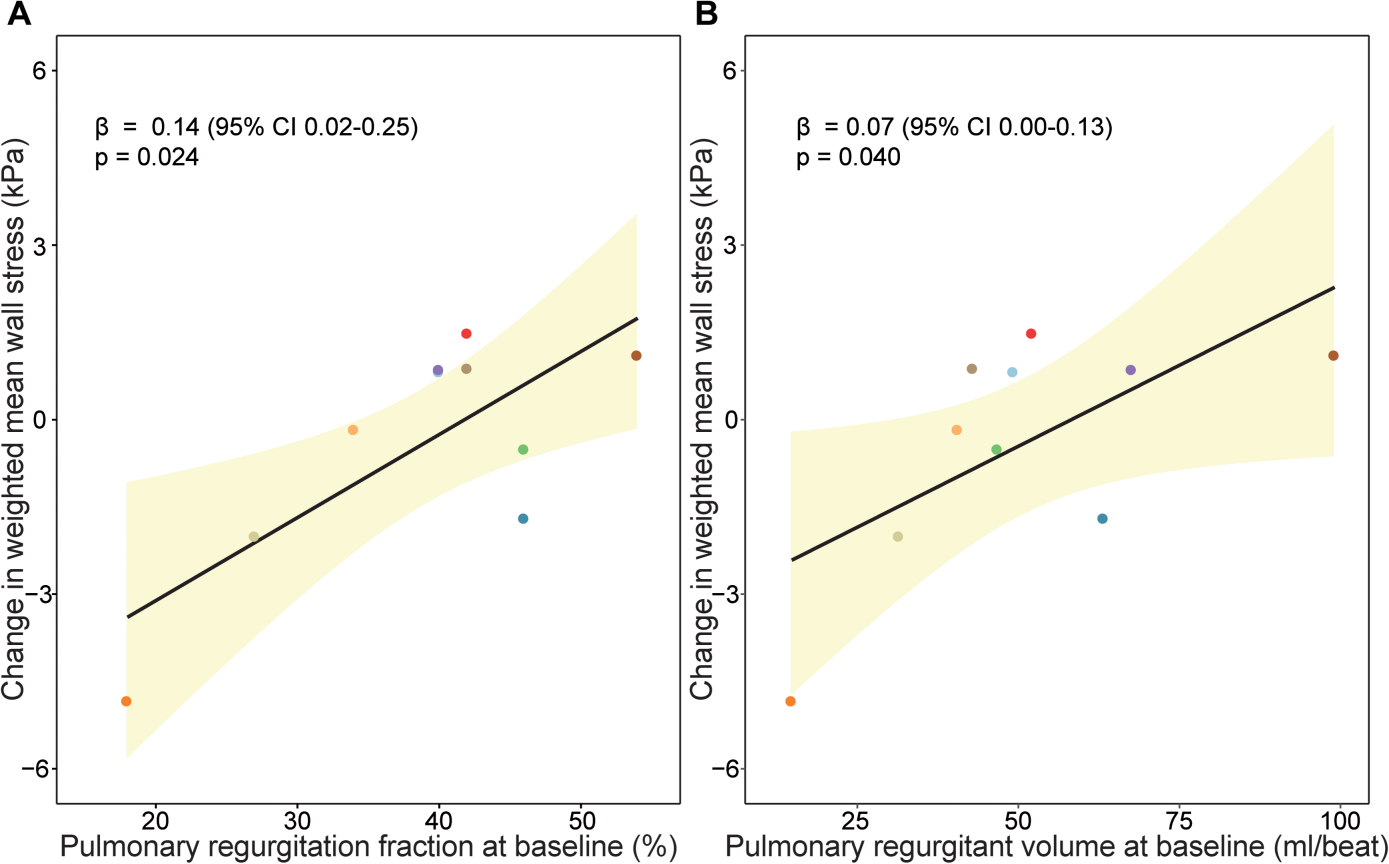
Association between pulmonary regurgitation and wall stress change over time. Association between (**A**) pulmonary regurgitation fraction (%) at baseline and (**B**) pulmonary regurgitant volume (ml/beat) at baseline and change in wall stress over time. Beta coefficients and *p*-value are visualized from linear mixed models and can interpreted as follows increase of wall stress of 0.14 kPa per percentage increase in pulmonary regurgitation fraction, *p* = 0.024, and 0.07 kPa increase of wall stress per ml pulmonary regurgitation volume increase, *p* = 0.040.

Finally, the relationship between PR and weighted mean wall stress was investigated (Figure 5). Patients with a higher PR fraction and a larger pulmonary regurgitant volume at baseline exhibited a larger increase of wall stress between baseline and follow-up when corrected for follow-up duration (increase of wall stress of 0.14 kPa per percentage increase in PR, *p* = 0.024, and 0.07 kPa increase of wall stress per ml PR volume increase, *p* = 0.040). In addition, patients with a decreasing RVEF had a larger PR volume than patients with a stable RVEF both at baseline and follow-up (Table 2). There was no significant change of PR and/or PR volume over time in patients with a stable or decreasing RVEF.

In accordance with the overall analysis, also baseline PR fraction was associated with regional changes in wall stress in the mid region β 0.21 (95% CI 0.04, 0.38), *p* = 0.025), anterior part β 0.11 (95% CI 0.03, 0.19), *p* = 0.015) and lateral part β 0.24 (95% CI 0.10, 0.39), *p* = 0.014) (Supplementary Figure S1). Also, PR volume at baseline was associated with regional wall stress changes in these regions (mid region β 0.11 (95% CI 0.04, 0.19), *p* = 0.010, anterior part β 0.05 (95% CI 0.01, 0.10), *p* = 0.033 and lateral part β 0.12 (95% CI 0.04, 0.19), *p* = 0.021).

## Discussion

In this study, we investigated changes of mechanical wall stress in the RV wall over time with computational modelling using standard clinical CMR- and echocardiography parameters. We applied this technique in the TOF population. We were able to show the inverse relationship of ventricular wall stress in the RV free wall with RV mass, mass-to-volume ratio and function *in-vivo*. Furthermore, RV wall stress decreased significantly over time in patients with a stable RV function, whereas RV wall stress did not decrease in patients with a decreasing RV function. Finally, patients with more PR at baseline had greater increase of their RV free wall stress over time.

These findings suggest that the method we used to calculate wall stress might be a useful addition in clinical decision making in TOF. Furthermore, it provides insight in the pathophysiological mechanisms behind RV adaptation to severe pulmonary valve regurgitation, RV enlargement and RV dysfunction in TOF patients. The computational methodology we used allows to study the mechanical status of the ventricular wall, including the regional differences throughout the right ventricle. Input for the model is derived from standard CMR, which is already the golden standard imaging modality to monitor volume, function and PR in TOF patients, and is routinely collected during follow-up at regular intervals. The current computational model can be improved, so that the current time to construct (±8 h) can be reduced until a clinically acceptable time.

Maintenance of adequate wall stress is a basic part of homeostasis in the ventricular wall. Previously, higher pre-operative ventricular wall stress has been associated with poorer PVR outcome in terms of RV function response to PVR in TOF patients (6). No studies are available on serial RV wall stress evaluation pre-PVR. In this study, in patients with at least moderate PR, ventricular wall stress decreased over time, particularly in patients that maintained their RVEF during follow-up, while in patients with deterioration of their RV function ventricular wall stress did not decrease. This suggests that wall stress might be a close marker of ventricular status than RVEF and RV volume assessment. Patients with a preserved RVEF increased their RV mass and RV mass-to-volume ratio. These are well-known mechanisms in ventricular adaptation to increased loading, aimed to normalized wall stress (19). To be able to recognize this process of wall stress adaptation in clinical practice, is, as stated, a potentially valuable tool.

In earlier studies, PR fraction and especially regurgitant volume has been associated with RV enlargement (20). Our findings suggest that the degree of PR has a direct effect on wall stress changes over time. Areas in which this relationship was most clear, were the mid and lateral part, which are located in the area of RV free wall most directly impacted by the pulmonary regurgitant flow jet. These regions correspond also to regions most prone to dilate (21). These studies and our findings suggest that PR might impact the ventricular wall locally and PR might be an important driver of wall stress increase.

Previous studies also indicate that PR is initially associated with more RV enlargement, mass-to-volume ratio decrease, thus resulting in higher wall stresses (22). However, this process is initially compensated by increasing the RV mass, keeping a relatively normal mass-to-volume ratio and preserving RV function (22). This was confirmed in the current study. Patients with a stable RVEF had less volume overload. In this group, there was an increase in mass and mass-to-volume ratio, while patients with a decreasing RVEF did not increase mass and mass-to-volume ratio. Consequently, these patients were not able to lower ventricular wall stress. The consequence of the lack of wall stress normalization is that myocardial perfusion could be impaired, resulting in a mismatch between blood flow supply and oxygen consumption. This could lead to decreased myocardial functioning, RV enlargement and ventricular wall thinning. RV mass-to-volume ratio was closely associated with wall stress, but was not associated RVEF changes over time.

Recently, attention has been drawn to RV geometric shape, which has been associated with exercise capacity, decrease in RV function and adverse events (23, 24). Wall stress is strongly depended on geometry. We hypothesize that these geometric adaptations might have led to unfavourable wall stress profile in the RV, thereby potentiates maladaptive remodelling and increases the risks on adverse events.

Strain, reflecting myocardial deformation, is a second mechanical parameter commonly measured. Previous studies suggest that a decrease of RV longitudinal strain and an increase RV circumferential strain in TOF patients compared to healthy controls (25). Both LV and RV strain have been associated with cardiovascular events (26). An unfavourable decrease of RV strain was observed in TOF patients over time (27). One study compared mechanical wall stress and strain, and this was around PVR. In that study, pre-operative wall stress was a better predictor of outcome after PVR than pre-operative strain (6). In this studies' models, the passive filling is modelled meaning stress and strain are coupled directly mathematically, therefore, strain values were not reported separately.

## Study limitations

The present study has several limitations. In our computational model, no specific material properties were implemented for the patch area and scar tissue. Pressures were estimated with echocardiography as invasive pressures were not available. The study consists of a small group of patients and, therefore, the results on the differences between patients with a stable and decreasing RVEF should be interpreted with care. Active contraction was not implemented in our model, consequently we could not analyse the systole. Apical contouring was hard due to the partial volume effect. Therefore, wall stress in this region should be interpreted with caution. Furthermore, the RV wall is thin and hard to accurately delineate given the spatial resolution. To improve accuracy and consistency, all contours were checked by a second experienced observer. Results of RV wall mass and mass-to-volume ratio were comparable to those previously reported (28). Current methodology is time consuming (±8 h) and analysis time should reduced for clinical implementation.

## Conclusion

Using computational modelling, RV mechanical wall stress was analysed in a regional manner in TOF patients. Wall stress was shown to closely relate to parameters such as RV mass, RV mass-to-volume ratio, PR and RVEF. This study demonstrates that RV wall stress can be determined based on routinely available input data and can be used in serial follow-up, providing a potential clinically relevant early marker of impending RV dysfunction.

## Data Availability

The raw data supporting the conclusions of this article will be made available by the authors, without undue reservation.
