# Supplementary material for: Serial RV wall stress measurements: association with right ventricular function in repaired Tetralogy of Fallot patients
Source: Front Cardiovasc Med. 2023 Oct 19;10:1256792. doi: 10.3389/fcvm.2023.1256792 (PMC10621746; doi:10.3389/fcvm.2023.1256792)

**Supplementary material**

| **Supplementary table 1 Regional wall stress changes over time stratified according to right ventricular function** | | | | | |
| --- | --- | --- | --- | --- | --- |
|  | **Total**  **(n=10)** | **Patients**  **with a stable RVEF**  **(n=6)** | **Patients**  **with a decreasing RVEF**  **(n=4)** | **β (95% CI) †** | **P-value †** |
| Anterior right ventricular free wall |  |  |  |  |  |
| Number of regions | 40 | 24 | 16 |  |  |
| Wall stress baseline (kPa) | 3.94 (2.64-5.87) | 4.41 (3.26-5.97) | 3.32 (2.07-5.32) | 0.47 (-0.31, 1.24)* | 0.199 |
| Wall stress follow-up (kPa) | 3.63 (2.61-5.05) | 3.25 (2.44-4.33) | 4.28 (3.07-5.95) | -0.38 (-0.87, 0.11)* | 0.107 |
| Change over time (kPa) | -0.33 ± 1.68 | -1.24 ± 1.62 | 0.83 ± 0.77 | -2.04 (-3.50, -0.58) | 0.029 |
| β (95% CI) ‡* | -0.27 (-0.58, 0.03) | -0.50 (-0.76, -0.25) | 0.40 (-0.30, 1.10) | - | - |
| P-value baseline vs FU ‡ | 0.082 | 0.004 | 0.255 | - | - |
|  |  |  |  |  |  |
| Lateral right ventricular free wall |  |  |  |  |  |
| Number of regions | 70 | 42 | 28 |  |  |
| Wall stress baseline (kPa) | 7.95 (4.86-13.00) | 9.24 (6.04-14.11) | 6.34 (3.82-10.53) | 0.62 (-0.32, 1.57)* | 0.166 |
| Wall stress follow-up (kPa) | 7.05 (4.37-11.37) | 6.83 (4.21-11.08) | 7.38 (4.58-11.89) | -0.19 (-0.73, 0.35)* | 0.513 |
| Change over time (kPa) | -1.01 ± 4.37 | -2.67 ± 4.96 | 1.11 ± 1.88 | -3.41 (-7.93, 1.11) | 0.118 |
| β (95% CI) ‡* | -0.42 (-0.72, -0.13) | -0.51 (-0.78, -0.23) | -0.47 (-1.30, 0.36) | - | - |
| P-value baseline vs FU ‡ | 0.005 | 0.004 | 0.261 | - | - |
|  |  |  |  |  |  |
| Posterior right ventricular free wall |  |  |  |  |  |
| Number of regions | 40 | 24 | 16 |  |  |
| Wall stress baseline (kPa) | 6.04 (4.05-9.00) | 6.51 (4.54-9.32) | 5.39 (3.47-8.39) | 0.31 (-0.37, 1.00)* | 0.510 |
| Wall stress follow-up (kPa) | 6.75 (4.74-9.61) | 6.73 (4.65-9.73) | 6.79 (4.84-9.52) | 0.02 (-0.56-0.60)* | 0.930 |
| Change over time (kPa) | 0.64 ± 2.52 | 0.25 ± 2.97 | 1.21 ± 1.56 | -1.15 (-3.50, 1.21) | 0.287 |
| β (95% CI) ‡* | 0.08 ( -0.19, 0.36) | 0.05 (-0.28, 0.38) | -0.04 (-0.68, 0.60) | - | - |
| P-value baseline vs FU ‡ | 0.558 | 0.756 | 0.907 | - | - |
| *Values are presented as numbers, mean and standard deviation (SD) or in case of a skewed distribution as geometric mean and geometric SD factor presented on the linear scale as geometric mean with range (geometric mean/geometric SD factor - geometric mean*geometric SD factor). Linear mixed effect models are created with the wall stress as dependent variable and timepoint (baseline or follow-up) as independent variable adjusted for time since initial surgery. Models have a random intercept per patient and a spatial Gaussian correlation structure. ‡ comparing baseline with follow-up. * Results are presented as the mean difference with 95% confidence interval (CI) of the wall stress expressed as 2log kPa. † comparing patients with a stable and a declining RVEF. CI = confidence interval, FU = follow-up, RVEF = right ventricular ejection fraction.* | | | | | |

| **RVEF (%)** | **Supplementary table 2 Multiple regression analysis** | | |
| --- | --- | --- | --- |
|  |  | **β (95% CI)** | **P-value** |
|  | *Model 1* | | |
|  | Weighted mean wall stress (kPa) | -1.27 (-2.36, -0.18) | 0.029 |
|  | LVEF (%) | 0.27 (-0.07, 0.61) | 0.103 |
|  | RVEDV indexed (ml/m^2^) | 0.01 (-0.10, 0.13) | 0.792 |
|  | Time since surgery (years) | -0.12 (-0.39, 0.14) | 0.305 |
|  | *Model 2* | | |
|  | RV Mass-to-volume ratio (g/ml) | 78.57 (-20.12, 177.26) | 0.099 |
|  | LVEF (%) | 0.24 (-0.13, 0.61) | 0.168 |
|  | RVEDV indexed (ml/m^2^) | 0.02 (-0.10, 0.15) | 0.679 |
|  | Time since surgery (years) | -0.08 (-0.37, 0.21) | 0.527 |
|  | *Model 3* |  |  |
|  | RV Mass-to-volume ratio (g/ml) | 63.92 (-22.11, 149.95) | 0.122 |
|  | LVEF (%) | 0.22 (-0.13, 0.57) | 0.185 |
|  | Time since surgery (years) | -0.07 (-0.33, 0.20) | 0.570 |
| *Linear mixed effect models are created with RVEF (%) as dependent variable. Models have a random intercept per patient. * β coefficient means for example that when the weighted mean wall stress increases with one unit RVEF decreased with 1.27% adjusted for LVEF, RVEDV and time since surgery. CI = confidence interval. LVEF = left ventricular ejection fraction, RV = right ventricular, RVEDV indexed = right ventricular end-diastolic volume indexed for body surface area.* | | | |

**Supplementary figure 1** Relationship between baseline pulmonary regurgitation fraction and weighted mean wall stress change over time depicted per region and part A) basal region B) mid region C) apical region D) anterior part E) lateral part F) posterior part


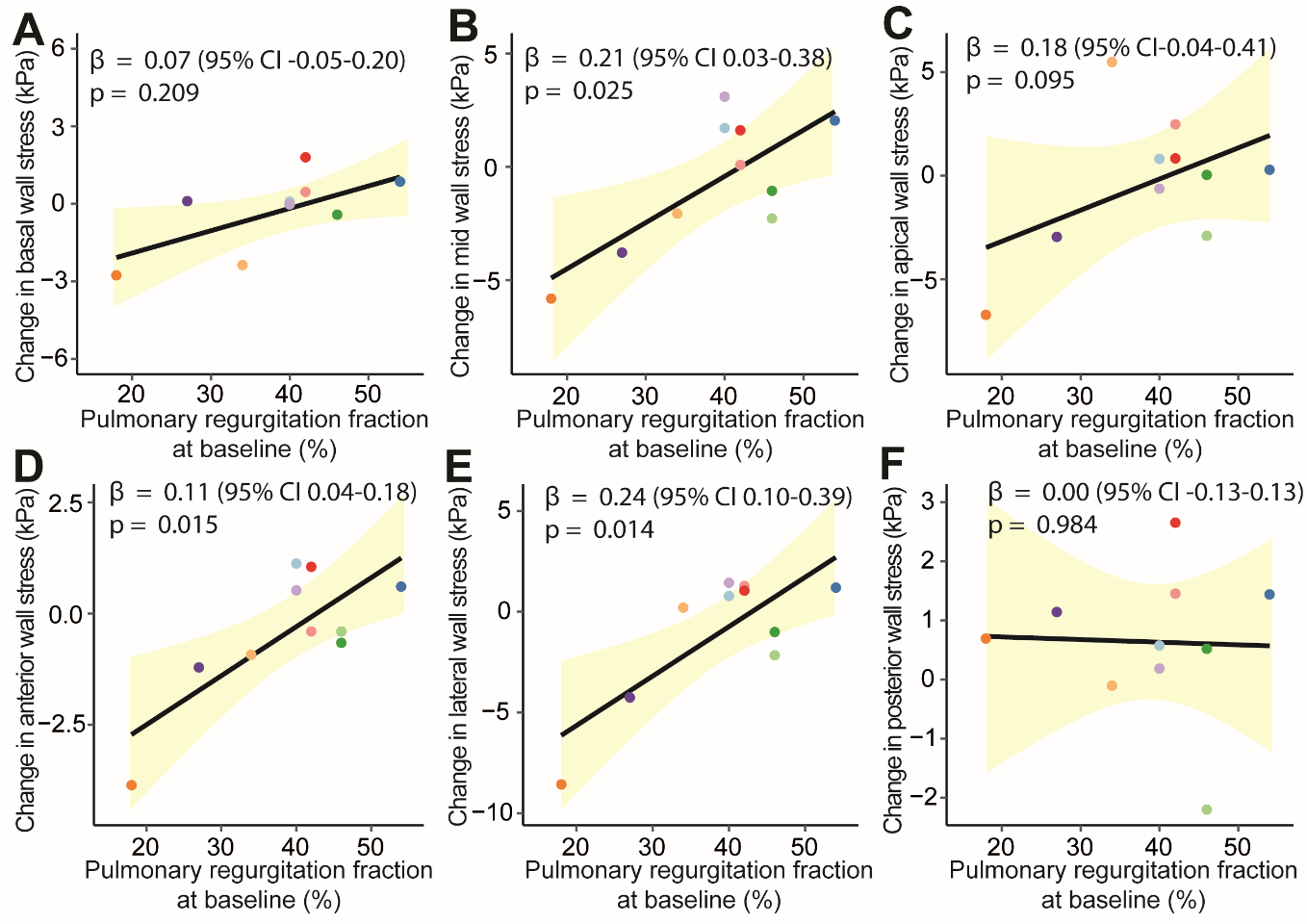

Supplement: Supplementary file 1 [file Table1.docx]
